# Complete genome sequence of *Microbulbifer* sp. CCB-MM1, a halophile isolated from Matang Mangrove Forest, Malaysia

**DOI:** 10.1186/s40793-017-0248-0

**Published:** 2017-07-06

**Authors:** Tsu Horng Moh, Nyok-Sean Lau, Go Furusawa, Al-Ashraf Abdullah Amirul

**Affiliations:** 1 Centre for Chemical Biology, Universiti Sains Malaysia, 11900 Penang, Malaysia; 2 School of Biological Sciences, Universiti Sains Malaysia, 11800 Penang, Malaysia

**Keywords:** Complete genome sequence, *Microbulbifer*, Halophile, Mangrove, Estuarine sediment

## Abstract

*Microbulbifer* sp. CCB-MM1 is a halophile isolated from estuarine sediment of Matang Mangrove Forest, Malaysia. Based on 16S rRNA gene sequence analysis, strain CCB-MM1 is a potentially new species of genus *Microbulbifer*. Here we describe its features and present its complete genome sequence with annotation. The genome sequence is 3.86 Mb in size with GC content of 58.85%, harbouring 3313 protein coding genes and 92 RNA genes. A total of 71 genes associated with carbohydrate active enzymes were found using dbCAN. Ectoine biosynthetic genes, *ectABC* operon and *ask_ect* were detected using antiSMASH 3.0. Cell shape determination genes, *mreBCD* operon, *rodA* and *rodZ* were annotated, congruent with the rod-coccus cell cycle of the strain CCB-MM1. In addition, putative *mreBCD* operon regulatory gene, *bolA* was detected, which might be associated with the regulation of rod-coccus cell cycle observed from the strain.

## Introduction

*Microbulbifer* sp. CCB-MM1 is a halophile isolated from an estuarine sediment sample taken from Matang Mangrove Forest, Malaysia. The genus *Microbulbifer* was proposed by González [1] with the description of *Microbulbifer hydrolyticus* which was isolated from marine pulp mill effluent. *Microbulbifer* are typically found in high-salinity environments including marine sediment [2], salt marsh [3], costal soil [4] as well as mangrove soil [5]. They were known for their capability to degrade a great variety of polysaccharides including cellulose [1, 5], xylan [1, 5, 6], chitin [1, 5, 6], agar [3, 6] and alginate [7]. *Microbulbifer* strains are potential sources of carbohydrate active enzymes with biotechnological interest. One of the species, *Microbulbifer mangrovi* had been reported with the ability to degrade more than 10 different polysaccharides [7].

Polysaccharides have a broad range of industrial applications. The most common storage polysaccharide, starch, can be used as food additives [8], excipients [9] and substrates in fermentation process to produce bioethanol [10]. Structural polysaccharides such as cellulose, chitosan and chitin, on the other hand, can be used to develop high-performance materials due to their renewability, biodegradability, biological inertness and low cost [11–13]. However, polysaccharides from natural sources are often not suitable for direct application. Chemical modifications involving the reactive groups (carboxyl, hydroxyl, amido, and acetamido groups) on the backbone of polysaccharide are required to alter their chemical and physical properties to suit the application purposes [14]. In the past years, explorations and researches are in favor of enzymatic method using carbohydrate active enzymes [15]. This alternative method offers the advantages of substrate specificity, stereospecificity, and environment friendly [16]. Hence, the discovery of novel carbohydrate active enzymes has great biotechnological interest and *Microbulbifer* strains are potential sources of these enzymes.

Therefore, we sequenced the genome of *Microbulbifer* sp. CCB-MM1 with primary objective to identify potential carbohydrate active enzyme coding genes. The genome insights will serve as baseline for downstream analyses including enzyme activity assays and functional elucidation of these genes. To date, there are seven genomes of *Microbulbifer* publicly available from GenBank, namely *Microbulbifer agarilyticus* S89 (NZ_AFPJ00000000.1) [17], *Microbulbifer variabilis* ATCC 700307^T^ (NZ_AQYJ00000000.1), *Microbulbifer elongatus* HZ11 (NZ_JELR00000000.1) [18], *Microbulbifer* sp. ZGT114 (LQBR00000000.1), *Microbulbifer thermotolerans* DAU221 (CP014864.1) [19], *Microbulbifer* sp. Q7 (LROY00000000.1) and *Microbulbifer* sp. WRN-8 (LRFG00000000.1). All of the *Microbulbifer* genomes are assembled to draft assembly only except the *Microbulbifer thermotolerans* DAU221 genome. Here we present the complete genome of *Microbulbifer* sp. CCB-MM1 and some insights from comparative analysis with seven other *Microbulbifer* genomes.

## Organism information

### Classification and features

**Fig. 1:**
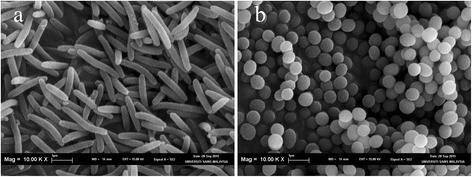
Scanning electron micrograph of *Microbulbifer* sp. CCB-MM1 at (**a**) exponential and (**b**) stationary phase

*Microbulbifer* sp. strain CCB-MM1 was isolated from mangrove sediment obtained from Matang Mangrove Forest. The isolation was done using the method previously described [20] with the use of H-ASWM (2.4% artificial sea water, 0.5% tryptone, 10 mM HEPES, pH 7.6) [21]. CCB-MM1 is a Gram-negative, aerobic, non-spore-forming and halophilic bacterium (Table 1). Its shape appears to be associated with its growth phases where it is rod-shaped at exponential phase (Fig. 1a) and cocci-shaped at stationary phase (Fig. 1b). The rod-shaped cell size ranges from approximately 1.3 to 2.5 μm in length and 0.3 μm in width while the diameter of coccus cells is approximately 0.6 μm. The colonies observed on agar plate are white in colour, circular, and raised with entire edge.

**Table 1:** Classification and general features of *Microbulbifer* sp. CCB-MM1 [69]

MIGS ID	Property	Term	Evidence code^a^
	Classification	Domain *Bacteria*	TAS [70]
		Phylum *Proteobacteria*	TAS [71]
		Class *Gammaproteobacteria*	TAS [72]
		Order *Cellvibrionales*	TAS [73, 74]
		Family *Microbulbiferaceae*	TAS [73, 74]
		Genus *Microbulbifer*	TAS [1]
		Species Unknown	IDA
		Strain CCB-MM1	IDA
	Gram stain	Negative	IDA
	Cell shape	Rod-coccus	IDA
	Motility	Non-motile	IDA
	Sporulation	Non-sporulating	NAS
	Temperature range	Mesophile	NAS
	Optimum temperature	30 °C	NAS
	pH range; Optimum	6.0–9.0; 7.0	IDA
	Carbon source	Not reported	
MIGS-6	Habitat	Estuarine sediment	IDA
MIGS-6.3	Salinity	Halophile	NAS
MIGS-22	Oxygen	Aerobic	IDA
MIGS-15	Biotic relationship	Free-living	NAS
MIGS-14	Pathogenicity	Non-pathogenic	NAS
MIGS-4	Geographic location	Malaysia: Matang Mangrove Forest	IDA
MIGS-5	Sample collection time	October 1, 2014	IDA
MIGS-4.1	Latitude	4.85228 N	IDA
MIGS-4.2	Longitude	100.55777 E	IDA
MIGS-4.3	Depth	10 cm	IDA
MIGS-4.4	Altitude	Not reported	

^a^Evidence codes - *IDA* inferred from direct assay, *TAS* traceable author statement (i.e., a direct report exists in the literature), *NAS* non-traceable author statement (i.e., not directly observed for the living, isolated sample, but based on a generally accepted property for the species, or anecdotal evidence). These evidence codes are from http://www.geneontology.org/GO.evidence.shtml of the Gene Ontology project [75]

**Fig. 2:**
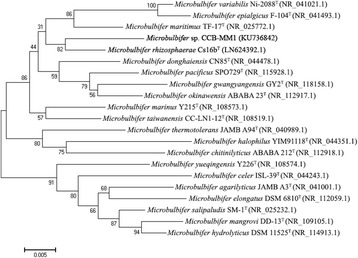
Neighbor-joining phylogenetic tree highlighting the position of *Microbulbifer* sp. CCB-MM1 relative to other type strains within the genus *Microbulbifer*, built using MEGA6 based on 16S rRNA sequences with their GenBank accession numbers indicated in parentheses

The 16S rRNA gene sequence of CCB-MM1 was amplified and sequenced using the universal primer pair 27F and 1492R [22]. The 16S rRNA gene sequence analysis was performed by using BLASTN [23] against NCBI 16S ribosomal RNA (Bacteria and Archaea) database. BLAST report revealed that the closely related strains include *Microbulbifer rhizosphaerae* Cs16b^T^ (98.1%), *Microbulbifer taiwanensis* CC-LN1-12^T^ (97.3%), *Microbulbifer maritimus* TF-17^T^ (97.4%), *Microbulbifer pacificus* SPO729^T^ (97.3%), and *Microbulbifer gwangyangensis* GY2^T^ (97.3%). Based on the threshold of *Proteobacteria* *-*specific 16S rRNA gene sequence similarity at 98.7% [24], the analysis suggests that CCB-MM1 is a new species belonging to the genus *Microbulbifer*. To reconstruct a phylogenetic tree of *Microbulbifer*, the 16S rRNA sequences of other *Microbubifer* type strains were downloaded from GenBank. Then, these sequences were aligned using MUSCLE [25, 26] and MEGA6 [27] was used to reconstruct a neighbour-joining tree [28] with 1000 replications of bootstrap method test [29]. As shown in Fig. 2, CCB-MM1 formed a cluster with *M. rhizosphaerae* Cs16b^T^ in the phylogenetic tree.

## Genome sequencing information

### Genome project history

Genome of CCB-MM1 was sequenced in October 2015. The whole genome sequencing and annotation were done by Centre for Chemical Biology (Universiti Sains Malaysia). The complete genome sequence is available in GenBank under the accession number CP014143. The project information is summarized in Table 2.

**Table 2:** Project information

MIGS ID	Property	Term
MIGS-31	Finishing quality	Complete
MIGS-28	Libraries used	PacBio P6-C4 chemistry, size selected 10 kb library, two SMRT Cells
MIGS-29	Sequencing platform	PacBio RS II
MIGS-31.2	Fold coverage	431×
MIGS-30	Assemblers	HGAP 3, PacBio SMRT Analysis v2.3
MIGS-32	Gene calling method	Prodigal
	Locus tag	AUP74
	Genbank ID	CP014143
	GenBank date of release	September 30, 2016
	GOLD ID	Gp0156207
	BIOPROJECT	PRJNA305828
MIGS-13	Source material identifier	SAMN04334609
	Project relevance	Environmental

### Growth conditions and genomic DNA preparation

CCB-MM1 was cultured aerobically in 100 mL of H-ASWM for overnight (16 h) at 30 °C with shaking. The genomic DNA was extracted using modified phenol-chloroform method [30]. The integrity of extracted genomic DNA was assessed by gel electrophoresis using 0.7% agarose gel and the quantification was done using NanoDrop 2000 Spectrophotometer (Thermo Scientific, USA).

### Genome sequencing and assembly

The whole genome of CCB-MM1 was sequenced using PacBio RS II platform with P6-C4 chemistry (Pacific Biosciences, USA). Two SMRT Cells were used and 2,674,097,380 pre-filter polymerase read bases were obtained, which was approximately 692X coverage of the genome. The reads were assembled using HGAP3 protocol [31] on SMRT Portal v2.3.0 with reads more than 25,000 bp in length being used as seed bases. The assembly result was a circular chromosome with the size of 3,864,326 bp, average base coverage of 431X and 100% base calling. The assembled sequence was polished twice using the resequencing protocol until the consensus concordance reached 100%.

### Genome annotation

The genome was annotated using Prokka 1.11 pipeline [32]. The pipeline uses Prodigal [33], RNAmmer [34], Aragorn [35], SignalP [36] and Infernal [37] to predict the coding sequences (CDS), ribosomal RNA genes, transfer RNA genes, signal leader peptides and non-coding RNAs, respectively. In addition, the translated CDS output by Prokka were used to BLAST against protein databases including non-redundant protein database (nr) from GenBank, Swiss-Prot and TrEMBL from UniProt [38], and KEGG database [39]. COG functional categories assignment was done using RPS-BLAST [40] search against the COG database [41]. In addition, antiSMASH 3.0 [42] was used to identify biosynthetic gene clusters and dbCAN [43] was used to identify carbohydrate active enzymes.

## Genome properties

**Fig. 3:**
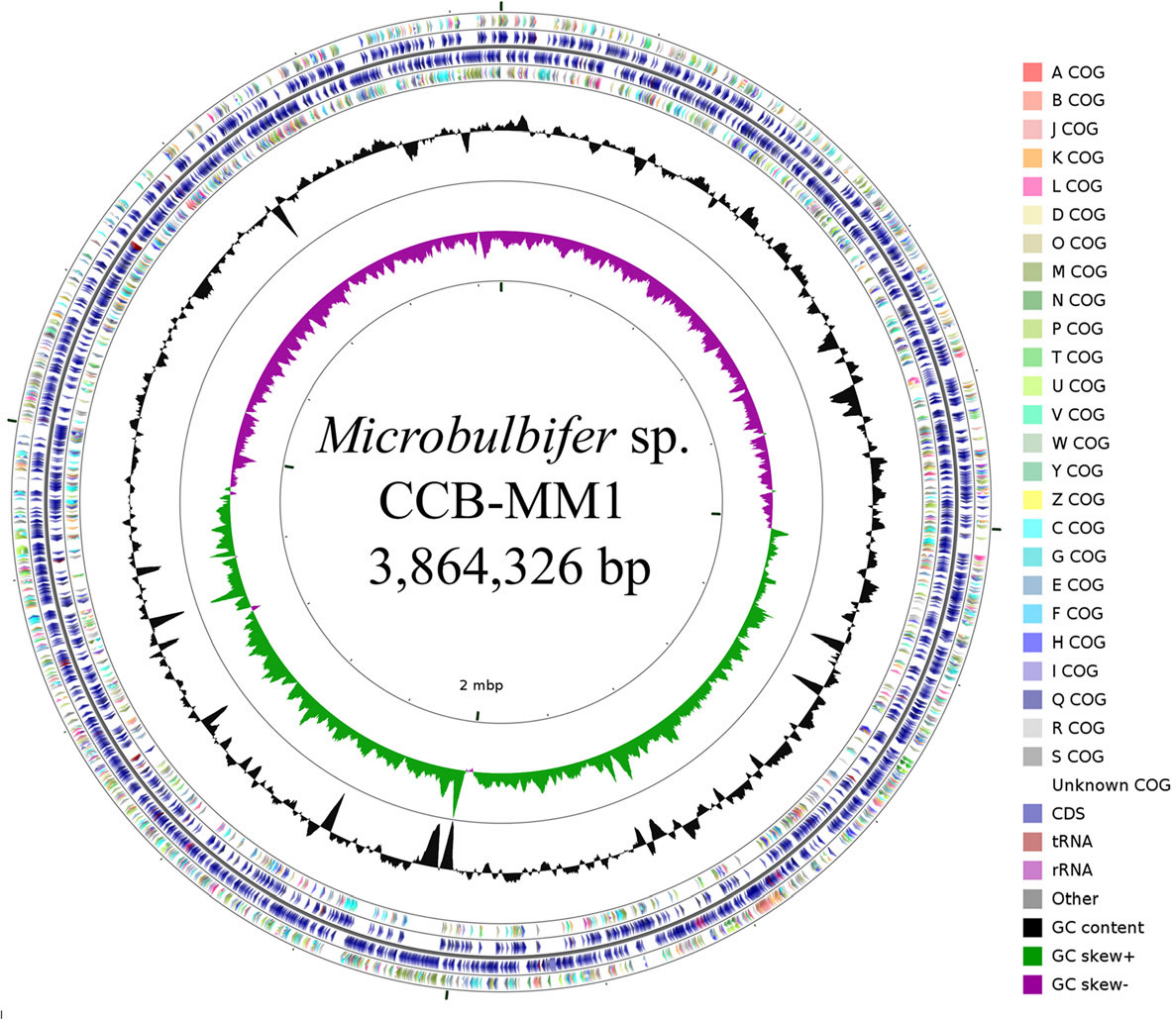
Circular map of the genome of *Microbulbifer* sp. CCB-MM1 generated using CGView Comparison Tool [44]. Circles (from outside) representing the following: 1. COG functional categories for forward coding sequence; 2. Forward sequence features; 3. Reverse sequence features; 4. COG functional categories for reverse coding sequence; 5. GC content; 6. GC skew

CCB-MM1 only contains one circular chromosome and no plasmid. The size of the chromosome is 3,864,326 bp with an overall of 58.85% G + C content (Table 3). The complete genome consists of 3313 ORFs, 79 tRNA, 12 rRNA and 1 tmRNA genes. Of all the 3313 predicted ORFs, 2030 of them can be assigned with functional prediction and 2563 of them can be assigned to COG functional categories (Table 4). The circular map of the genome generated using CGView Comparison Tool [44] is depicted in Fig. 3.

**Table 3:** Genome statistics

Attribute	Value	% of Total^a^
Genome size	3,864,326	100.00
DNA coding (bp)	3,487,727	90.25
DNA G + C (bp)	2,274,198	58.85
DNA scaffolds	1	-
Total genes	3406	100.00
Protein coding genes	3313	97.27
RNA genes	92	2.70
Pseudo genes	1	0.03
Genes in internal clusters	-	-
Genes with function prediction	2030	59.62
Genes assigned to COGs	2563	75.27
Genes with Pfam domains	2856	83.88
Genes with signal peptides	403	11.84
Genes with transmembrane helices	851	24.99
CRISPR repeats	0	0

^a^The total is based on either the size of the genome in base pairs or the total number of protein coding genes in the annotated genome

**Table 4:** Number of genes associated with general COG functional categories

Code	Value	% age^a^	Description
J	229	6.9	Translation, ribosomal structure and biogenesis
A	2	0.1	RNA processing and modification
K	127	3.8	Transcription
L	111	3.3	Replication, recombination and repair
B	0	0.0	Chromatin structure and dynamics
D	41	1.2	Cell cycle control, cell division, chromosome partitioning
Y	0	0.0	Nuclear structure
V	64	1.9	Defense mechanisms
T	109	3.3	Signal transduction mechanisms
M	218	6.6	Cell wall/membrane/envelope biogenesis
N	8	0.2	Cell motility
Z	2	0.1	Cytoskeleton
W	3	0.1	Extracellular structures
U	48	1.4	Intracellular trafficking, secretion, and vesicular transport
O	173	5.2	Posttranslational modification, protein turnover, chaperones
X	3	0.1	Mobilome: prophages, transposons
C	180	5.4	Energy production and conversion
G	131	4.0	Carbohydrate transport and metabolism
E	212	6.4	Amino acid transport and metabolism
F	53	1.6	Nucleotide transport and metabolism
H	113	3.4	Coenzyme transport and metabolism
I	133	4.0	Lipid transport and metabolism
P	167	5.0	Inorganic ion transport and metabolism
Q	55	1.7	Secondary metabolites biosynthesis, transport and catabolism
R	226	6.8	General function prediction only
S	224	6.8	Function unknown
-	751	22.7	Not in COGs

^a^The total is based on the total number of protein coding genes in the annotated genome

## Insights from the genome sequence

### Comparative genomics

**Fig. 4:**
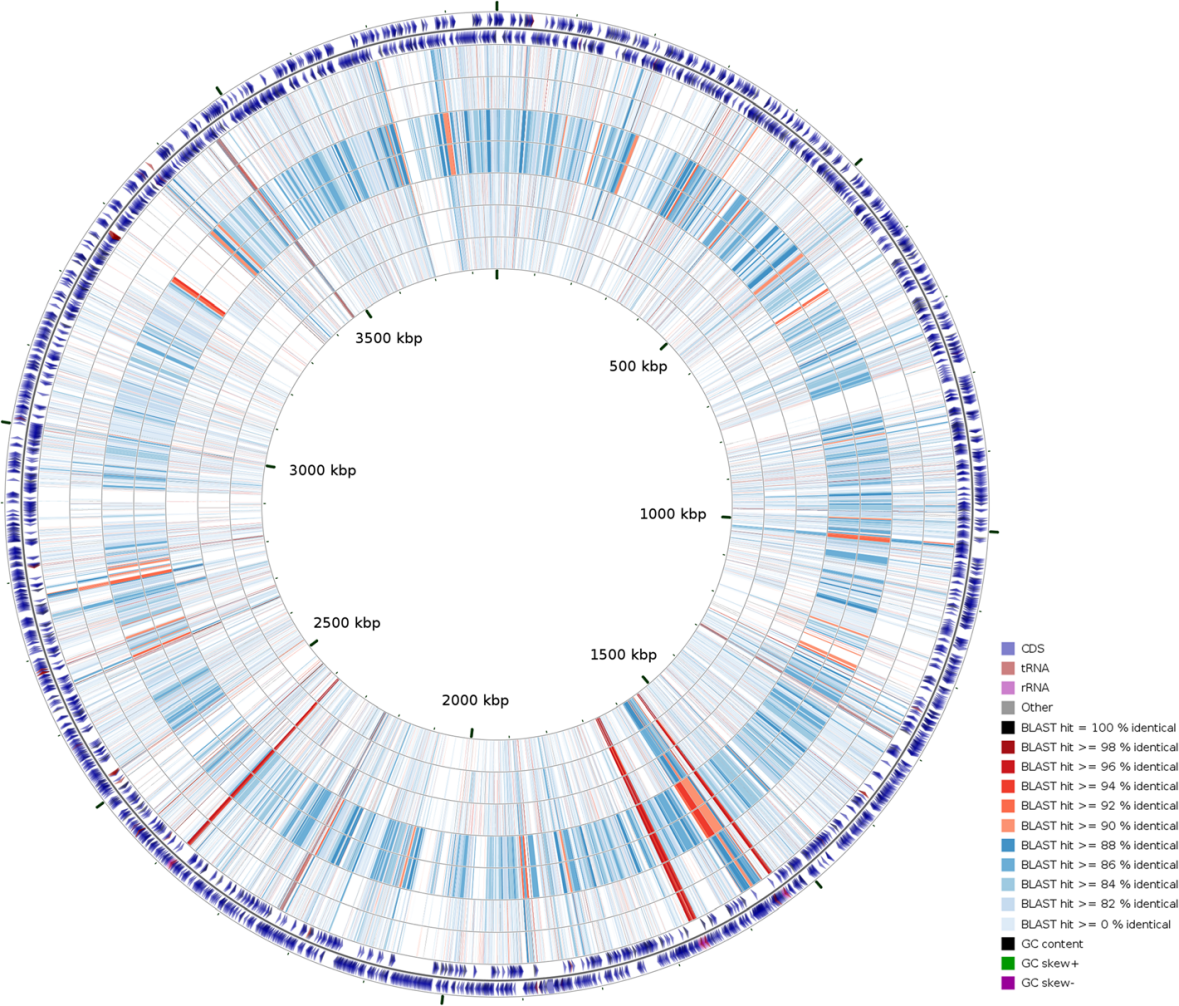
Circular map comparing strain CCB-MM1 genome and seven other *Microbulbifer* genomes generated using CGView Comparison Tool [44]. The two outermost rings represent forward and reverse sequence features respectively. The remaining seven rings show the regions of sequence similarity detected by BLAST comparisons conducted between nucleotide sequences from the CCB-MM1 genome and seven other *Microbulbifer* genomes with the order (from outside) as follow: *Microbulbifer elongatus* HZ11, *Microbulbifer* sp. Q7, *Microbulbifer* sp. WRN-8, *Microbulbifer* sp. ZGT114, *Microbulbifer agarilyticus* S89, *Microbulbifer thermotolerans* DAU221 and *Microbulbifer variabilis* ATCC 700307^T^

There are seven genomes of *Microbulbifer* strains publicly available in GenBank to date. To assess the relatedness between CCB-MM1 and publicly available *Microbulbifer* genomes, ANI values between the genomes were calculated using method based on MUMmer alignment [45]. Based on the results (Table 5), the ANI values ranged from 85.58% (*Microbulbifer* sp. ZGT114 and *Microbulbifer* sp. WRN-8) to 83.45% (*Microbublfer thermotolerans* DAU221). These ANI values fall below 95% [46], suggesting that CCB-MM1 represents a different species from the other seven sequenced species. Interestingly, the ANI value between genomes of *Microbulbifer* sp. ZGT114 and *Microbulbifer* sp. WRN-8 is 99.99%, which suggests that these two strains belong to the same species. The circular map comparing CCB-MM1 genome and seven other *Microbulbifer* genomes is shown in Fig. 4.

**Table 5:** ANI value(%) between *Microbulbifer* sp. CCB-MM1 genome and seven other *Microbulbifer* genomes calculated using ANIm [45]

	CCB-MM1	ZGT114	WRN-8	HZ11	S89	Q7	ATCC 700307^T^	DAU221
CCB-MM1	100.00	85.58	85.58	84.75	84.65	84.61	84.37	83.45
ZGT114	85.58	100.00	99.99	84.65	84.64	84.70	84.29	83.85
WRN-8	85.58	99.99	100.00	84.65	84.70	84.67	84.29	83.87
HZ11	84.75	84.65	84.65	100.00	85.23	85.58	84.68	83.71
S89	84.65	84.64	84.70	85.23	100.00	85.03	84.77	83.66
Q7	84.61	84.70	84.67	85.58	85.03	100.00	84.75	83.77
ATCC 700307	84.37	84.29	84.29	84.68	84.77	84.75	100.00	83.59
DAU221	83.45	83.85	83.87	83.71	83.66	83.77	83.59	100.00

CCB-MM1 = *Microbulbifer* sp. CCB-MM1; ZGT114 = *Microbulbifer* sp. ZGT114; WRN-8 = *Microbulbifer* sp. WRN-8; HZ11 = *Microbulbifer elongatus* HZ11; S89 = *Microbulbifer agarilyticus* S89; Q7 = *Microbulbifer* sp. Q7; ATCC 700307^T^ = *Microbulbifer variabilis* ATCC 700307^T^; DAU221 = *Microbulbifer thermotolerans* DAU221

### Carbohydrate active enzymes

dbCAN [43] was used to predict carbohydrate-active enzyme coding genes present in CCB-MM1 genome, particularly genes belonging to glycoside hydrolase and polysaccharide lyase families that could provide us the insights on carbohydrate degrading capability of CCB-MM1. The analysis was done by running HMMER3 [47] scan using HMMs profile downloaded from dbCAN (version: dbCAN-fam-HMMs.txt.v4) with an *e*-value cut off of 1*e*-18 and coverage cut off of 0.35. A total of 71 carbohydrate-active genes were detected and further analysis of these genes using SignalP predicted that 25 of them contain signal peptides. As shown in Table 6, we had found 29 genes associated with GH families including GH3, GH5, GH13, GH16, GH20, GH23, GH31, GH38, GH103 and GH130, however, we found no genes associated with PL families in the genome. Annotation of the GH genes revealed that CCB-MM1 genome possesses genes encoding cellulase (GH5), alpha-amylase, pullulanase (GH13) and beta-glucanase (GH16) with potential interest for biotechnological applications. While gene coding for beta-hexosaminidase, one of the chitinolytic enzymes [48], is present in the genome of CCB-MM1, gene that codes for chitinase was not detected. This suggests that CCB-MM1 lacks the ability to degrade chitin, although further assays are required to confirm the phenotype.

**Table 6:** GH enzyme coding genes found in CCB-MM1 genome

GH Family	Annotation	Signal peptide	Locus tag
3	Periplasmic beta-glucosidase precursor	Yes	AUP74_01723
	Periplasmic beta-glucosidase precursor	No	AUP74_01724
	Beta-hexosaminidase	No	AUP74_02396
	Beta-hexosaminidase A precursor	Yes	AUP74_02833
5	Cellulase (glycosyl hydrolase family 5)	No	AUP74_03275
	hypothetical protein	No	AUP74_03276
13	Pullulanase precursor	Yes	AUP74_00304
	Oligo-1,6-glucosidase	No	AUP74_00394
	Cyclomaltodextrinase	Yes	AUP74_00399
	4-alpha-glucanotransferase	No	AUP74_00401
	Alpha-amylase precursor	Yes	AUP74_00413
	Sucrose phosphorylase	No	AUP74_03226
16	Glucan endo-1,3-beta-glucosidase A1 precursor	No	AUP74_01725
	Beta-glucanase precursor	Yes	AUP74_01727
20	N,N′-diacetylchitobiase precursor	No	AUP74_01890
23	Membrane-bound lytic murein transglycosylase F precursor	Yes	AUP74_00546
	Membrane-bound lytic murein transglycosylase F precursor	No	AUP74_01553
	Membrane-bound lytic murein transglycosylase F precursor	Yes	AUP74_01554
	murein transglycosylase C	Yes	AUP74_01596
	Membrane-bound lytic murein transglycosylase D precursor	Yes	AUP74_02266
	Soluble lytic murein transglycosylase precursor	Yes	AUP74_02385
	Membrane-bound lytic murein transglycosylase F precursor	No	AUP74_03185
	Membrane-bound lytic murein transglycosylase F precursor	No	AUP74_03186
	Membrane-bound lytic murein transglycosylase F precursor	Yes	AUP74_03326
31	Alpha-xylosidase	Yes	AUP74_00400
38	Mannosylglycerate hydrolase	No	AUP74_01043
103	Membrane-bound lytic murein transglycosylase B precursor	Yes	AUP74_01186
	Membrane-bound lytic murein transglycosylase B precursor	Yes	AUP74_01707
130	4-O-beta-D-mannosyl-D-glucose phosphorylase	No	AUP74_03278

### Rod-coccus cell cycle

*Microbulbifer* were found to demonstrate rod-coccus cell cycle, in association with different growth phases [49]. This cell cycle was also observed in CCB-MM1. In CCB-MM1 genome, we found genes which are known to be involved in determining and maintaining the rod shape of bacteria, including *mreBCD* [50] (AUP74_00016, AUP74_00017 and AUP74_00018), *rodA* [51] (AUP74_01706) and *rodZ* [52] (AUP74_01850). BLAST analysis showed that these genes are present in all other *Microbulbifer* genomes. In addition, we detected the presence of general stress response gene, *bolA*, in all *Microbulbifer* genomes. It has been demonstrated that the overexpression of *bolA* in *E.coli* inhibited cell elongation and reduced the transcription of *mreBCD* operon [53]. The gene, *mreB*, and its product, actin homolog have been studied for their functions in several species of bacteria. This protein lies beneath the cell surface, forming actin-like cables which function as guidance for the synthesis of longitudinal cell wall [54]. While MreB is not essential in *E. coli* [55], it is found to be essential for *Streptomyces coelicolor* [56], *Rhodobacter sphaeroides* [57] and *Bacillus subtilis* [58]. In *E. coli*, depletion of MreB caused cells to change from rod-like to spherical shape but these cells were able to survive [59]. In contrast, the spherical-shaped *B. subtilis* cells eventually lyse. For CCB-MM1, the spherical-shaped cells do not lyse but grow into rod-shaped again after being transferred into fresh medium. We infer that *mreB* gene may have important functions in determining *Microbulbifer* cell shape and the rod-coccus cycle of *Microbulbifer* is likely regulated by BolA through inhibition of *mreB* transcription when triggered by stress.

### Secondary metabolites, ectoine

Ectoine and hydroxyectoine are compatible solutes found primarily in halophilic bacteria. When triggered by osmotic stress, bacteria produce and accumulate them intracellularly to balance the osmotic pressure [60]. Apart from osmotic stress, they were also protectants against temperature stress [61]. A cluster of genes responsible for the biosynthesis of ectoine [62] has been identified in CCB-MM1 genome using antiSMASH 3.0 [42]. These genes encode for aspartate kinase (Ask_Ect) (AUP74_00280), _L_-ectoine synthase (EctC) (AUP74_00281), diaminobutyrate-2-oxoglutarate transaminase (EctB) (AUP74_00282), _L_-2,4-diaminobutyric acid acetyltransferase (EctA) (AUP74_00283) and HTH transcriptional regulator (AUP74_00284). The lack of the gene *ectD*, ectoine hydroxylase, in CCB-MM1 genome suggests that it only has the ability to synthesize ectoine but not hydroxyectoine. By using BLASTP, we searched and found similar gene cluster in other *Microbulbifer* genomes except *Microbulbifer variabilis* ATCC 700307 ^T^. While the reason for the absence of these genes in *Microbulbifer variabilis* ATCC 700307 ^T^ is unknown, our findings suggest that *Microbulbifer* utilized only ectoine instead of ectoine/hydroxyectoine mixture. The transcriptional regulator of ectoine operon, EctR, found in *Methylophaga thalassica* belongs to MarR family [63]. HTH transcriptional regulator (AUP74_00284) in CCB-MM1 also contains the conserved domain of MarR family. This implies that the HTH transcriptional regulator is likely the putative transcriptional regulator of ectoine operon in *Microbulbifer*. Ectoine has attracted considerable biotechnological interest due to its stabilizing effects that extend from proteins [64], nucleic acids [65] to whole cells [66]. Such properties allow it to be used in skin care product as cell protectants [66], protein stabilizers [67] and medical application as cryoprotectants in cryopreservation of human cells [68].

## Conclusion

In this study we presented the complete genome sequence of *Microbulbifer* sp. CCB-MM1 with genome size of 3.86 Mb and G + C content of 58.85%. We discussed some insights on its phenotypic characteristics from the genomic perspective, covering carbohydrate active enzymes, rod-coccus cell cycle and secondary metabolite, ectoine. The genome sequence provides valuable information for functional elucidations of novel enzymes for both biotechnological application and fundamental research purposes.

## Abbreviations

ANI: Average nucleotide identity
antiSMASH: Antibiotics & Secondary Metabolite Analysis Shell
CCB: Centre for Chemical Biology
dbCAN: Database for automated carbohydrate-active enzyme annotation
GH: Glycoside hydrolase
H-ASWM: High nutrient artificial seawater media
MM: Matang Mangrove
PL: Polysaccharide lyase
